# Applying machine learning methods to enable automatic customisation of knee replacement implants from CT data

**DOI:** 10.1038/s41598-023-30483-5

**Published:** 2023-02-27

**Authors:** Thomas A. Burge, Jonathan R. T. Jeffers, Connor W. Myant

**Affiliations:** 1 Dyson School of Design Engineering, Imperial College, London, SW7 2BU UK; 2 Department of Mechanical Engineering, Imperial College, London, SW7 2BU UK

**Keywords:** Biomedical engineering, Mechanical engineering

## Abstract

The aim of this study was to develop an automated pipeline capable of designing custom total knee replacement implants from CT scans. The developed pipeline firstly utilised a series of machine learning methods including classification, object detection, and image segmentation models, to extract geometrical information from inputted DICOM files. Statistical shape models then used the information to create femur and tibia 3D surface model predictions which were ultimately used by computer aided design scripts to generate customised implant designs. The developed pipeline was trained and tested using CT scan images, along with segmented 3D models, obtained for 98 Korean Asian subjects. The performance of the pipeline was tested computationally by virtually fitting outputted implant designs with ‘ground truth’ 3D models for each test subject’s bones. This demonstrated the pipeline was capable of repeatably producing highly accurate designs, and its performance was not impacted by subject sex, height, age, or knee side. In conclusion, a robust, accurate and automatic, CT-based total knee replacement customisation pipeline was shown to be feasible and could afford significant time and cost advantages over conventional methods. The pipeline framework could also be adapted to enable customisation of other medical implants.

## Introduction

Total Knee Replacement (TKR) procedures involve removing the damaged articular surfaces of a patient’s knee joint and implanting femoral and tibial components to alleviate pain and restore kinematics. To achieve a high quality fit and minimise post-procedure complications, TKR implant components can be customised for individuals, especially when subjects are observed to have abnormal anatomies^1^. Typically, to obtain the geometrical information necessary to design unique components, 3D medical imaging, such as Computerised Tomography (CT) imaging, is utilised^1,2^. Such methods produce 3D images comprising of thousands of 2D ‘slices’, usually in the form of ‘Digital Imaging and Communication in Medical’ (DICOM) files^3^. To generate 3D models of subjects’ anatomies, a laborious and time consuming manual segmentation process is then required^4^. This, combined with a bespoke manual design process for each individual’s implant designs, consequently means custom TKR procedures incur longer lead times, higher costs and are far less commonly used than non-customised ‘off-the-shelf’ alternatives^5^.

In related studies, authors have outlined how machine learning techniques, such as Convolutional Neural Networks (CNNs), can be used to automate the segmentation of CT scans and expedite the generation of 3D models for structures including the skull^6^ and the spine/vertebrae^7,8^. CNNs have also been shown compatible with Magnetic Resonance Imaging (MRI) data. For example, Prasoon et al. exhibited how knee cartilage can be segmented from scans automatically by adopting the technology^9^. Moreover, authors have outlined how the design of custom medical devices, including cranial implants^6,10^ and ocular prostheses^11^, can be achieved automatically from CT data via similar techniques. Burge et al. used various machine learning techniques to develop a ‘fully automated mass-customisation pipeline’ for producing TKR components from bi-planar X-ray images^12^. The authors however found that, despite multiple benefits of using X-rays as input (reduced radiation exposure, cost, imaging time etc.), to facilitate quality outputs, precise calibration and tightly controlled positioning/alignment were required. Furthermore, slightly poorer performance was reported for the X-ray-based pipeline compared to other 3D imaging-based methods, particularly for geometrically complicated femur components^13^.

Although numerous approaches for developing custom TKR implant components using 3D medical imaging data (CT and MRI) have been outlined in the literature^1,3,14,15^, all these methodologies required substantial manual input and failed to address the aforementioned cost and lead time burdens. This study therefore outlines a new approach for automating CT-based custom TKR implant design. Machine learning-based medical image processing methods are exploited to enable a ‘mass-customisation’ pipeline—the automated design of customised products for the masses^12^. The ambition is that such a solution would enable customised implants to be fabricated faster and with lower cost, making the numerous potential benefits of customisation^16,17^ more obtainable. The proposed, proof-of-concept pipeline is outlined with the various models trained to process CT scans, extract subject specific information, and create 3D model predictions detailed. The method is then validated by testing its performance on 84 subject femur and 81 tibia bones.

## Methods and pipeline development

### Data sets and test subjects

A data set comprising 98 subjects was obtained from the Korea Institute of Science and Technology Information (KISTI) for training and testing the pipeline^18^. The data set featured CT scans with a resolution of 0.832 × 0.832 mm, 1 mm slice thickness (axial field of view) and image size of 512 × 512 pixels. 3D models, created by segmenting the CT data, were supplied with the scans for subjects’ left and right femur and tibia bones. The subjects comprised of Korean Asians with an even split of males and females. The subjects ranged from 21 to 60 years old and 146 to 176 cm in height. The subjects were split into two approximately equal sized groups for training and testing the automatic implant customisation pipeline. Where possible, data for both knees for each subject was used. In certain cases, subjects’ data was found to be missing or of poor quality (CT scans with poor contrast and alignment, and/or 3D models with erroneous acute holes and/or extrusions). These files were removed from the data set. In total, data from 51 subjects (33 female and 28 male) was used for training the pipeline. This consisted of slice images from 43 CT scans, as well as 90 femur and 96 tibia 3D models (including both left and right bones). Test subjects were selected only if patients’ CT scans and 3D bone models were available and of good quality. A separate 45 subjects (20 female and 25 male) were used to test the performance of the pipeline for the femur with 43 subjects (22 female and 21 male) used for the tibia.

All human data used in this manuscript was retrospective and sourced from the KISTI dataset. Ethical approval and consent were obtained by KISTI who also ensured the data was appropriately anonymised before distribution. The study protocol and associated ethics were reviewed and approved by the Research Governance and Integrity Team at Imperial College, in line with relevant guidelines and regulations. Reference number 6423670.

### CT scan to 3D model prediction

**Figure 1:**
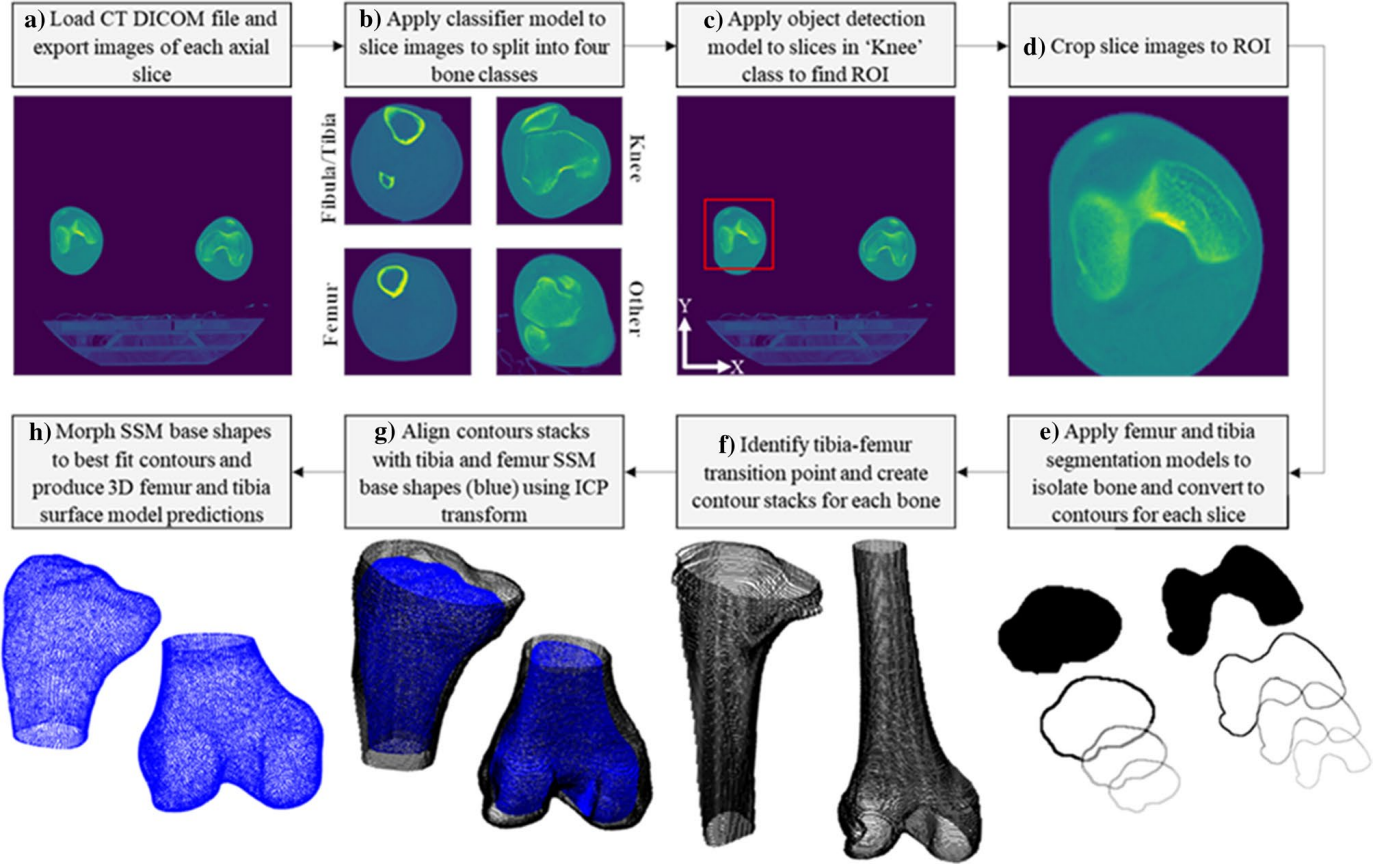
Workflow of CT-3D surface model prediction process. (**a**) Load DICOM, (**b**) classify slices, (**c**) identify ROI, (**d**) crop slices to ROI, (**e**) segment femur and tibia bones, (**f**) create contour stacks, (**g**) align contour stacks with SSM base shapes, (**h**) morph SSMs to fit contour points and create 3D model predictions.

For the pipeline to produce customised implant components, 3D predictions of subjects’ femur and tibia anatomies were created. A top-level overview of the CT scan to 3D model workflow, written using Python 3, is illustrated in Fig. 1.

Firstly, the inputted CT DICOM files were loaded into the pipeline and information such as pixel resolution, slice spacing, and number of slices was recorded. Each slice from the CT scan was exported and saved as individual ‘.PNG’ files (Fig. 1a).

A classifier model was applied to the slice images to split them into four classes—‘Fibula/Tibia’, ‘Knee’, ‘Femur’, and ‘Other’. The ‘Knee’ class comprised images of the condylar region of the distal femur, proximal tibia, and patella bones (Fig. 1b). A deep CNN architecture, similar to that outlined by Hou and Gao^19^, was utilised for the classifier model. 8000 slice images (2000 for each class) were categorised and utilised for training the model with 800 (200 for each class) reserved for validation. After classifying the CT slices the bone order was determined by the pipeline (tibia–knee–femur or femur–knee–tibia).

An object detection model was applied to each slice determined to be within the ‘Knee’ region to locate the Region of Interest (ROI) around the left knee (Fig. 1c). A ‘bounding box regression’ object detection model was utilised with a CNN architecture similar to that outlined by Galvez et al.^20^. To train the model, 1000 training images, labelled with bounding box coordinates around the left knee, were used with 100 reserved for validation. After the centre of the ROI was determined for each slice, a 160 × 160 pixel square box, positioned around the median X and Y coordinates, was defined as the average ROI across all slices. Each slice image in the ‘Knee’ class was then cropped to this region and outputted as a new .PNG file (Fig. 1d). If an implant design for the right knee was required, the slice images were flipped via a horizontal transformation in the pipeline before the object detection step was completed.

To isolate the femur and tibia bones from the surrounding tissue in the cropped ROI images, segmentation models for both bones were applied (Fig. 1e). Using models trained for each bone (as opposed to one for both) enabled more accurate geometrical information to be obtained. The segmentation models utilised a ‘U-Net’ CNN architecture based on Ronneberger et al.^21^. To train the models, 1832 and 1623 training image/mask pairs were generated for the femur and tibia respectively. These featured CT slices cropped to the ROI around the left knee, as well as the right after a horizontal transformation had been applied. This helped to increase the volume of training data available. 200 image/mask pairs were reserved for validation for each model. In the pipeline, both segmentation models were applied to all ‘Knee’ slices before a Canny edge detector was employed to extract the bone contours as shown in Fig. 1e. To remove possible segmentation noise, a filtering process was used to eliminate lines below a specified length and retain only the bone contours from each slice.

The classification, object detection and segmentation models described above were built using TensorFlow^22^ and trained using slice images obtained from the 43 CT scans reserved for training. The hyperparameters of each model (learning rate, number of epochs, batch size, etc.) were adjusted to achieve optimal performance on the designated validation data before being tested on new data in the full pipeline.

**Figure 2:**
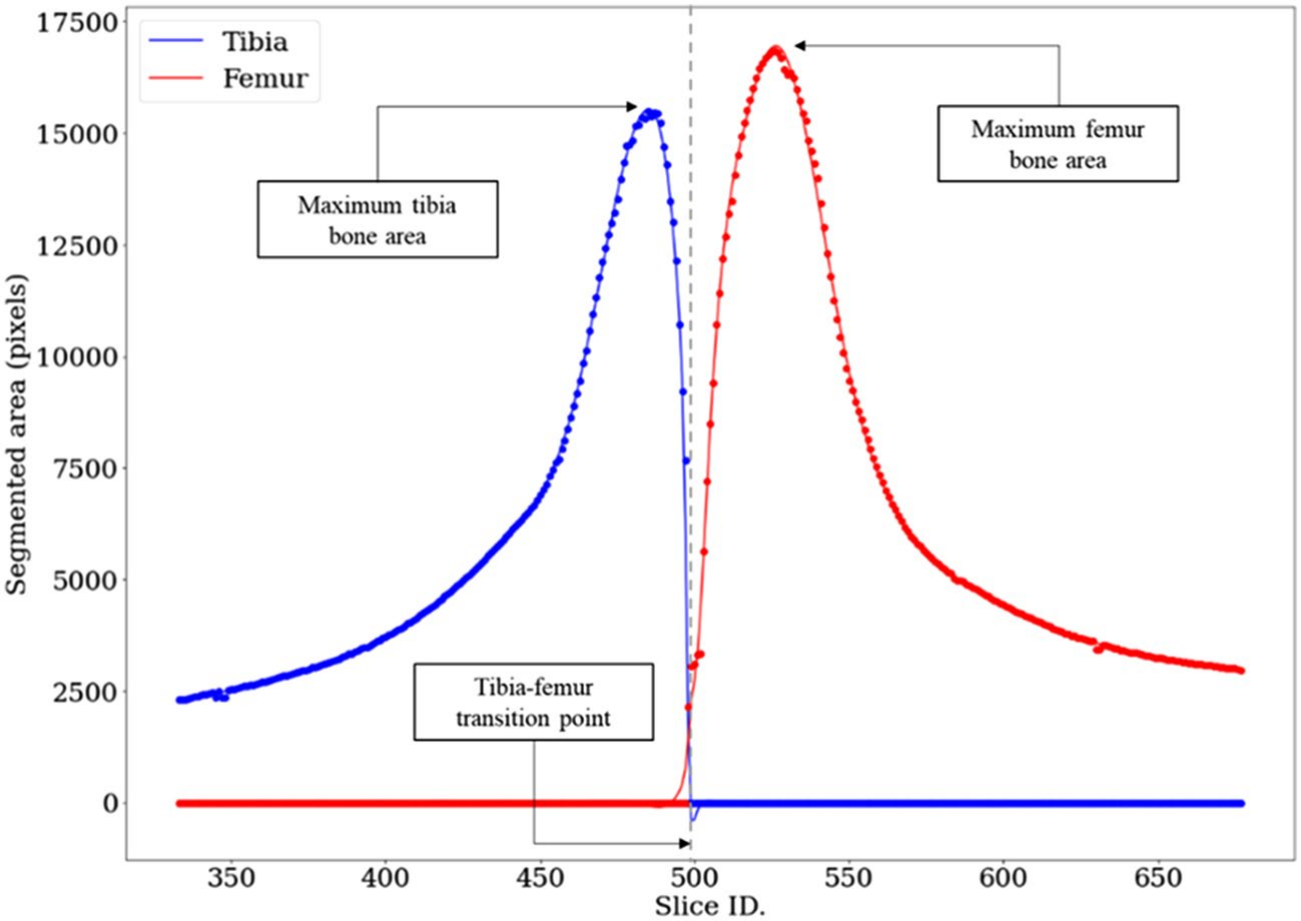
Tibia and femur segmented areas by slice. Plot shows segmented bone areas plotted for each slice within the ‘Knee’ region of a CT stack. Dashed line indicates the identified tibia–femur transition point.

To identify the tibia–femur transition point and separate the contours, firstly, the area of each slice’s segmented area (for both femur and tibia segmentation model results) was calculated as illustrated in Fig. 2. The transition point was then determined by locating the position of the largest area for both the femur and tibia bones in the slices and finding the slice at which the tibia and femur curves intersected between these reference points. This is where the condyles of the femur end and the tibia plateau begins and was found to be a reliably identifiable reference point. To form a ‘contour stack’ for the tibia, the 2D contours produced using the tibia segmentation model, up to the defined tibia–femur transition point, were positioned on top of each other in 3D space. The contours were separated in the Z direction (normal to the slices) according to slice spacing information retrieved from the inputted CT DICOM file. The same was completed to form femur contour stacks using contours generated by the femur segmentation model after the tibia–femur transition point. The contour stacks were trimmed by five slices at both the proximal end of the tibia and the distal end of the femur as excessive segmentation noise was found to occur in these regions. Example contour stacks for both bones are illustrated in Fig. 1f.

Two Statistical Shape Models (SSMs)—one for each bone, were developed. To train the models, code was utilised from Nolte et al.^23^ that analyses the variation in geometry across a population of 3D models and reduces the dimensionality down to a controllable number of ‘principal components’. As an example, these could include the spacing between femur condyles or the tibia plateau angle. Further detail on the development of SSMs for use with knee X-ray images is outlined in^24,25^. The SSMs developed for the femur and tibia in this study were trained using 90 and 96 3D models respectively. These comprised of left bones, as well as right bones after horizontal transformations were applied. To create 3D predictions of subjects’ bones, the contour stacks generated were aligned to the base shapes of each SSM model using a ridged Iterative-Closest-Point (ICP) method (Fig. 1g). An optimisation algorithm was then used to morph the SSM base shapes to best fit the contour stacks and produce 3D surface model predictions (Fig. 1h). To obtain accurate global predictions, but ignore imperfections such as osteophytes and holes, the number of principal components used to morph the SSMs was limited to two. Furthermore, the SSM base shapes were ‘idealised’ and smoothed so outputted models were suitable for use in designing custom implants. This approach is consistent with Burge et al.^12,13^.

### Custom implant design

**Figure 3:**
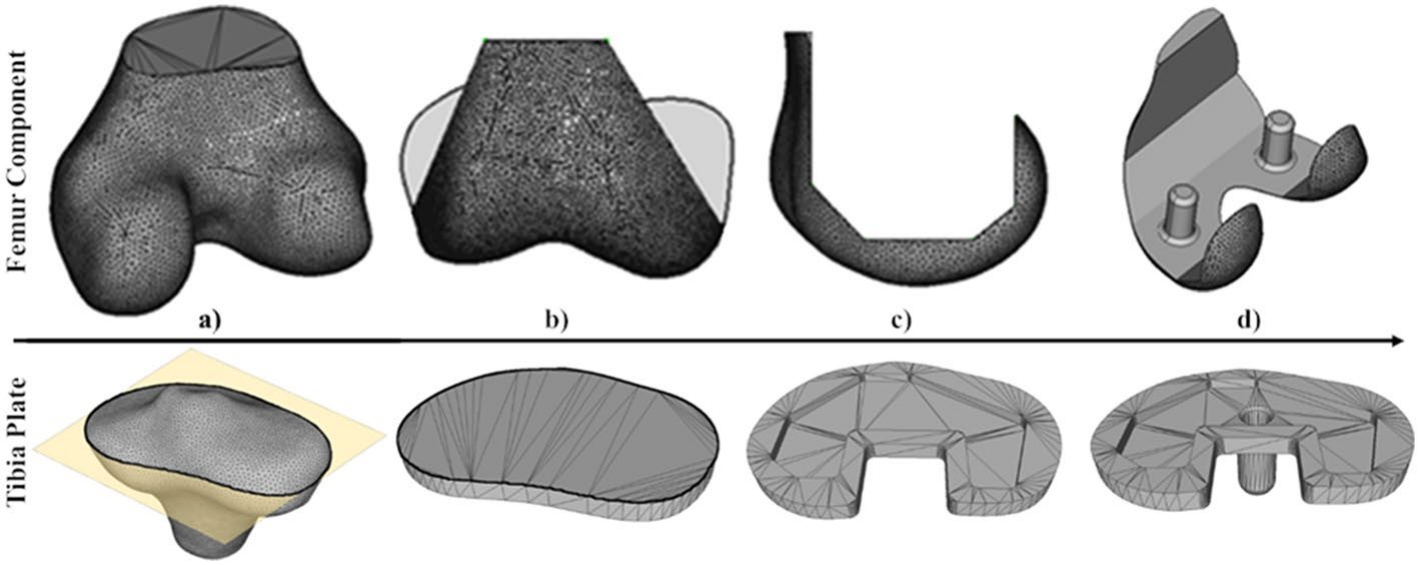
Automatic custom femur component (top row) and tibia plate (bottom row) design process. Femur—(**a**) load 3D model prediction, (**b**) add anterior–posterior cuts, (**c**) add medial–lateral cuts, (**d**) add pins, fillets, and chamfers. Tibia—(**a**) extract 2D profile from 3D model prediction, (**b**) extrude profile, (**c**) add bearing connection and posterior cuts, (**d**) add pin, fillets, and chamfers.

Figure 3 illustrates the major stages of the generic TKR femur component (top row) and tibia plate (bottom row) design process, enabled via Application Programming Interface (API) Python scripts interfacing with the software FreeCAD^26^. For femur components, subjects’ 3D surface model predictions were converted to solid bodies (Fig. 3a). Then predefined operations were applied to cut the model to the implant shape in both the anterior–posterior (Fig. 3b), and medial–lateral (Fig. 3c) views. Finally, features such as fixation pins were extruded, and fillets/chamfers added (Fig. 3d). The operations were scaled to work for individuals based on maximum condylar anterior–posterior and medial–lateral measurements captured by the pipeline. To design tibia plates, a 2D cross-section was taken 2 mm below the widest point of the medial condyle on each subject’s 3D model predictions from a plane created parallel with the surface of the tibia plateau^13^ (shown in Fig. 3a). This was utilised as the base profile for the component and was extruded to a thickness of 5 mm (Fig. 3b). Features were cut for interfacing with a polyethylene bearing component and at the posterior of the plate (Fig. 3c). A fixation pin was then extruded, and fillets/chamfers added (Fig. 3d).

### Performance metrics

Three metrics were used to evaluate pipeline performance. The accuracy of the CT-3D model predictions was assessed by calculating the surface-surface Root Mean Squared Error (RMSE) of subjects’ model predictions compared to reference models. For this analysis, the 3D segmented models supplied with the KISTI data set were regarded as ground truth. The RMSE calculation was performed after aligning the two models in 3D space using a rigid ICP method. Equation (1) details the RMSE calculation performed where N was the number of points and ${x}_{i}-{\widehat{x}}_{i}$ was the Euclidian distance between points on the two model surfaces within the condylar regions.

1 $$RMSE= \sqrt{\frac{{\sum }_{i=1}^{N}{({x}_{i}-{\widehat{x}}_{i})}^{2}}{N}}$$

**Figure 4:**
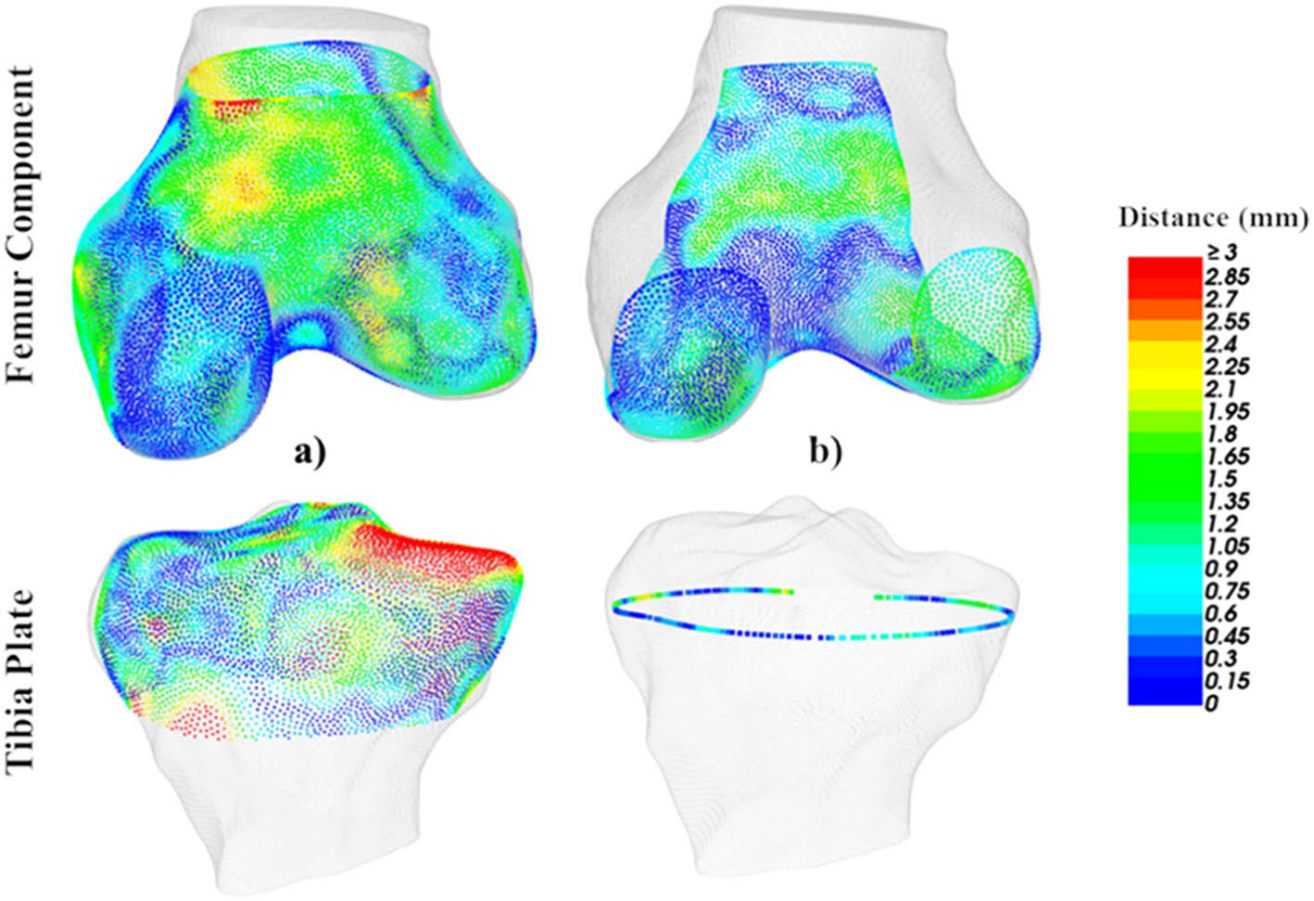
Distance heat maps for the femur (top row) and tibia (bottom row). (**a**) CT-3D model predictions and (**b**) custom components, both compared to ground truth models.

The femur and tibia plate components generated by the pipeline were also aligned to the ground truth models via rigid ICP transformations and RMSEs calculated. For femur components, the 3D surface of the implant (without fixation pins) was used. For tibia plates, the RMSE calculation was performed between the 2D base profile extracted from the 3D model prediction (like in Fig. 3a), and an equivalent profile taken at the same point on the ground truth model. This approach was used for the tibia plate as it only interfaces with the resected tibia bone on one face. The 3D surface model prediction and component to ground truth RMSE calculations are illustrated in Fig. 4 as distance heatmaps.

**Figure 5:**
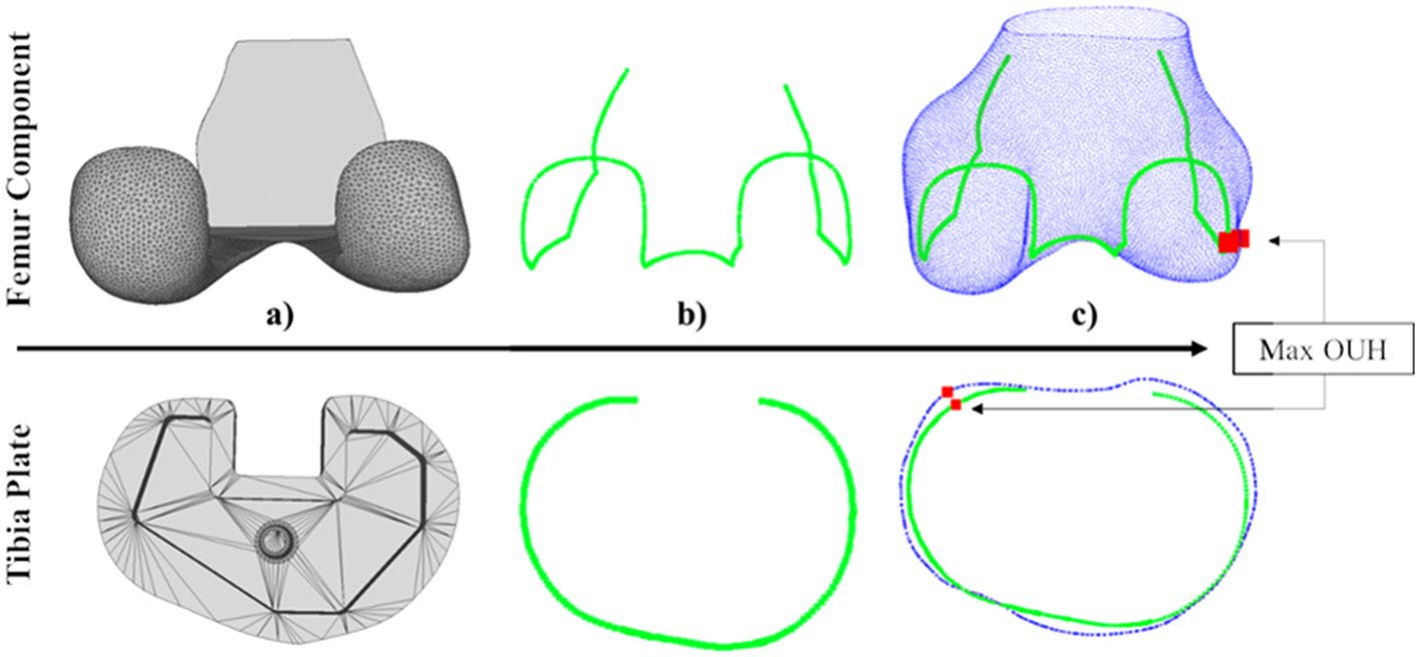
Calculation of the maximum OUH for the femur component (top row) and tibia plate (bottom row). (**a**) Implant components, (**b**) component edges, (**c**) component edges compared to ground truth geometry and identification of maximum OUH.

To provide a more clinical fit metric, the maximum Over or Under Hang (OUH) between the edges of custom implant components and the bone after resection was evaluated. Excessive OUH has been shown to lead to complications post-surgery, such as tissue irritation and instability. Generally, ≥ 3 mm OUH is regarded as significant and used as a threshold^27,28^. In this analysis, maximum OUH was reported as the Hausdorff distance (h) anywhere between the edges of the component (C) and the edges of the resected bone (B), with the distance (d) between each point pairing (c and b) calculated as the Euclidian distance. The calculation is described by Eq. (2) and illustrated in Fig. 5.

2 $$h(C,B)= {max }_{\mathrm{c }\in {\mathrm{ C}}} \{ {min}_{{\mathrm{ b} }\in {\mathrm{ B}}} \{ d(c,b)\}\}$$

### Statistical analysis

For statistical analysis, two-sample t-tests were utilised to evaluate differences in means between test subject attribute pairs, such as the performance of males vs. females. *T* tests were adopted over analysis of variance (ANOVA) as there is a lower chance of error and only a limited number of attribute pairs were assessed. Data was confirmed to be approximately normally distributed using quantile–quantile plots and outliers were removed before performing statistical tests. p values ≤ 0.05 were considered statistically significant throughout.

To evaluate the correlation strength of continuous variables on performance, such as subject age or height, Spearman's correlation coefficients were calculated. Spearman’s coefficients ≤ − 0.5 or ≥ 0.5 were considered significant.

## Results

**Figure 6:**
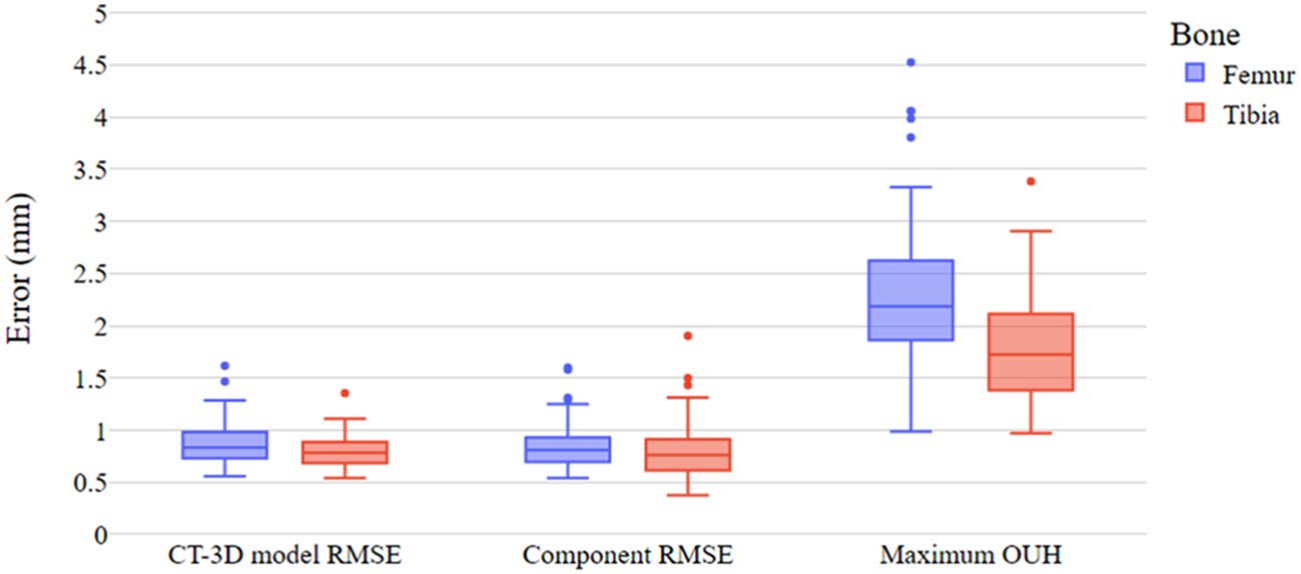
Box plot of pipeline results. Results split into three metrics: 3D model prediction RMSE, component RMSE and maximum OUH.

The pipeline successfully generated 3D femur and tibia predictions and implant components for all subjects, except for one tibia. In this case, the pipeline failed to segment the CT slices and generate the necessary contour stacks to create the 3D model predictions. This test subject (both left and right knees) was subsequently removed from the tibia analysis leaving 42 subjects (81 bones). The pipeline performance is detailed in Table 1 for both bones. The table includes mean values for the 3D model prediction and component to ground truth RMSE, as well as the number of test subject knees resulting in a maximum OUH above the clinically significant threshold (≥ 3 mm). The three performance metrics for the femur and tibia are also illustrated graphically in Fig. 6. The plot shows a limited number of outliers (data points located outside the box plot whiskers) were recorded for both bones across the three performance metrics. It was found that, typically, these isolated cases of poor fit were due to imperfect segmentation results.

**Table 1:** Pipeline performance for the femur and tibia. Results split into three metrics: 3D model prediction RMSE, component RMSE, and component maximum OUH ≥ 3.

	Total test subject bones	3D model prediction mean RMSE (mm), (sd)	Component RMSE mean (mm), (sd)	Component maximum OUH ≥ 3 mm, (%)
Femur	84	0.87 (0.20)	0.85 (0.21)	6 (7.1)
Tibia	81	0.80 (0.15)	0.82 (0.27)	1 (1.2)

Comparing results for the femur (n = 84) and tibia (n = 81) showed significant differences for 3D model prediction RMSE (p = 0.017) and maximum OUH (p << 0.001). A significant difference was however not calculated between the femur and tibia in terms of component RMSE (p = 0.39). Within the results for each bone, the difference in performance due to sex and knee side was not found to be statistically significant for any of the three performance metrics (p values all > 0.05). Additionally, neither subject age nor height were found to significantly impact the pipeline performance for both bones as all Spearman’s coefficients calculated (r) were − 0.1 < r < 0.3.

In terms of solve time, the pipeline was found to consistently process inputted CT DICOM files and generate both custom femur and tibia plate components in less than 10 min when run on a personal laptop computer (2.38 GHz AMD Ryzen 5 4500U CPU with 6 cores/8 GB memory). The majority of the solve time was incurred whilst processing the CT DICOM files and generating the contour stacks. These stages were required regardless of the desired pipeline out, meaning minimal time savings where observed for subjects requiring just one component type as opposed to both.

## Discussion

This study outlined a machine learning-based, fully automatic pipeline for processing CT scans and designing custom TKR implant components. An analysis of the results obtained for 84 subject femurs and 81 tibias demonstrated the pipeline to be capable of repeatably producing highly accurate results. Implant designs were produced in less than 10 min, which, combined with the benefit of removing laborious manual CT scan segmentation and implant design, could afford significant cost and time savings over current customisation solutions. Furthermore, highly trained users would not be required to operate the pipeline.

The pipeline performance was found to be better for constructing tibia plate designs compared to femur components when evaluated in terms of maximum OUH. This was likely due to the higher complexity of the femur morphology and the component interfacing with the bone over multiple faces. The performance of the CT-based pipeline was substantially better for both bones across all three metrics compared to that reported for the X-ray-based tool outlined by Burge et al.^13^. The CT-based pipeline would also not be affected by X-ray related errors such as subject orientation/alignment and magnification effects. Nevertheless, requiring CT data as an input would increase costs, limit accessibility for the technology and increase patient radiation exposure. In comparison to results reported for other CT-based custom TKR solutions, Ogura et al. completed a study featuring 55 patients (59 knees) fitted with ConforMIS ‘Bicompartmental’ custom implants, concluding OUH ≥ 3 mm was not present for any subject^29^. Likewise, Arnholdt et al. reported no instances of OUH ≥ 3 mm in a study featuring 91 patients fitted with customised ConforMIS ‘iTotal™ CR G2’ implants^30^. Contrary to these, Schroeder and Martin evaluated 44 patients implanted with ConforMIS ‘iTotal CR’ implants, concluding 18% had tibia plates implanted with OUH ≥ 3 mm^16^. It should be noted that in^29,30^ maximum OUH was measured post-operatively via 2D X-rays and defined as the distance between the outermost edges of the tibia/femoral condyles and the outermost edges of the component. This differs to the method utilised in this study which considered the entire bone-implant interface and likely resulted in higher levels of OUH being recorded than would have been if a similar method to^29,30^ was adopted. The results published by Schroeder and Martin^16^, who also measured OUH along the entire bone-implant interface, are therefore a more relevant comparison. Hence, it can be concluded that the automated pipeline performance could be comparable, or possibly better, than non-automated alternatives.

In one instance, the pipeline failed to process a subject’s CT scan and produce 3D model predictions of their tibia anatomy. Moreover, a small number of outliers (where poor fits were observed for both the femur and tibia processes) were recorded. These issues stemmed from erroneous results produced by the segmentation models, likely caused by overfitting in the machine learning and statistical models as a consequence of the limited data available for training. Although a reasonably large number of images were obtained to train the image-based models, these were sourced from just 43 CT scans captured via the same equipment and were purely of Korean Asian subjects. For training the SSMs, approximately 100 3D models were used for each bone. However, these consisted of left and right bones from the same subjects, consequentially reducing learnable variation. To mitigate this, and better generalise the pipeline, a more diverse data set including subjects from a range of ethnicities, ages, etc., imaged using multiple CT scanners with various resolutions, should be utilised. Furthermore, subject attributes including sex, knee side, height and age were demonstrated not to significantly impact the performance of the pipeline. However, due to the data available, it was not possible to test the impact of subject characteristics such as ethnicity and/or arthritis severity on performance. Identifying a larger, more diverse data set would also enable a more comprehensive sensitivity study to be completed for the pipeline and its limitations better verified.

To introduce the automated pipeline in practice, the workflow developed in this study could be adapted to include a software interface for clinicians to use easily without knowledge of coding languages. Through this, patient CT data could be uploaded, along with implant design requirements, and the pipeline could run remotely on the cloud. Once implant designs are ready, they could be sent directly by the software to a third party to be produced and sent back to clinicians, ready for surgery. In the literature, various articles have detailed how fabrication of personalised implants can be achieved in biocompatible materials, such as titanium, via Additive Manufacturing (AM). AM techniques such as metal Selective Laser Sintering (SLS) are said to be overtaking the traditional machining and casting processes for implant manufacture as they can accelerate production, achieve better specifications, and reduce costs^31^. Furthermore, authors have also argued that by using customised implants, the overall cost of knee replacement procedures can be reduced further by minimising surgery time, revision rates and implant storage^2^. To bring the automated pipeline to market, the most significant challenge would likely be the associated regulatory requirements. For example, regulators may require each patient’s custom implant designs to be evaluated by a trained medical professional and/or engineer before surgery. This could potentially diminish the cost, resource and lead time advantages gained from the automatic workflow.

In this study, the performance of the novel pipeline was assessed using purely computational methods; no prototype models of the custom implant components designed were fabricated and no clinical work was completed. Therefore, factors such as poor surgical implementation were not considered as part of the fit analysis. Results could have also been impacted by inaccuracies in the segmented 3D bone models provided with the KISTI data set. Future work could look to use more accurate methods for obtaining ground truth data, such as hand scanning dissected bones, and clinical experimentation could be completed. The practical limitations of implementing an automated pipeline for designing custom implants should be investigated further, as well as the potential limitations incurred by requiring AM as a fabrication method. Finally, although generic TKR components were used in this study, it would be possible to adapt the pipeline to work with commercially available designs. Applying the CT-based pipeline framework to other medical implants, such as uni-condylar knee replacements^12^, hip replacements^32^, or for predicting the best non-customised TKR implant sizes^33^, could also be explored.

## Conclusion

In this study, a proof-of-concept pipeline was developed that utilised various machine learning methods to enable automatic data extraction from CT DICOM files, produce 3D model predictions of patients’ anatomy and design customised TKR implants. The proposed workflow was shown to be repeatable, accurate and generated results rapidly without any user input required. It is also flexible and could be adopted for different implant designs and types. Nevertheless, to fully validate the proposed pipeline, it should be trained and tested more thoroughly using additional subject data and via both computational and clinical methods.

## Data Availability

The data that support the findings of this study are available from the Korea Institute of Science and Technology, but restrictions apply to the availability of these data, which were used under license, and so are not publicly available. Data are however available from the authors upon reasonable request and with permission of the Korea Institute of Science and Technology.
